# Strong Decrease in Streptomycin-Resistance and Absence of XDR 12 Years after the Reorganization of the National Tuberculosis Control Program in the Central Region of Cameroon

**DOI:** 10.1371/journal.pone.0098374

**Published:** 2014-06-05

**Authors:** Larissa Kamgue Sidze, Emmanuel Mouafo Tekwu, Christopher Kuaban, Jean-Paul Assam Assam, Jean-Claude Tedom, Sara Eyangoh, François-Xavier Fouda, Désiré Nolna, Francine Ntoumi, Matthias Frank, Véronique N. Penlap Beng

**Affiliations:** 1 Laboratory for Tuberculosis Research (LTR), Biotechnology Center (BTC), University of Yaoundé I, Yaoundé, Cameroon; 2 Pneumology Unit, Jamot Hospital, Yaoundé, Cameroon; 3 Mycobacteriology Service, Reference laboratory of NTP, Centre Pasteur of Cameroon-Pasteur Institute International Network, Yaoundé, Cameroon; 4 Mbalmayo District Hospital, Mbalmayo, Cameroon; 5 National Tuberculosis Control Program (NTCP), Yaoundé, Cameroon; 6 Fondation Congolaise pour la Recherche Médicale, Université Marien Ngouabi, Brazzaville, Congo; 7 Institute of Tropical Medicine, University of Tübingen, Tübingen, Germany; 8 Deutsches Zentrum für Infektionsforschung (DZIF), Tübingen, Germany; 9 Central African Network for Tuberculosis, AIDS/HIV and Malaria (CANTAM), Brazzaville, Congo; Institute of Infectious Diseases and Molecular Medicine, South Africa

## Abstract

**Background:**

In the 1990s, resistance rates of 15% for streptomycin-resistance and 0.6% for multidrug-resistance (MDR) were reported from the Central Region of Cameroon. This work assesses drug resistant tuberculosis in this region 12 years after reorganization of the National Tuberculosis Control Program (NTCP).

**Methods:**

This cross-sectional study was conducted from April 2010 to March 2011 in Jamot Hospital in Yaoundé, Cameroon. Only patients with smear positive pulmonary tuberculosis were included. Sputa were cultured and subsequently underwent drug susceptibility testing (DST). All consenting individuals were tested for their HIV status.

**Results:**

A total of 665 smear positive pulmonary tuberculosis patients were enrolled. The HIV prevalence was 28.5% (95%CI [25.2–32.1]). Of the 582 sputa that grew *Mycobacterium tuberculosis* complex species, DST results were obtained for 576. The overall resistance rate was 10.9% (63/576). The overall resistance rates for single drug resistance were: isoniazid-resistance 4.7% (27/576), streptomycin-resistance 3.3% (19/576), rifampicin-resistance 0.2% (1/576), kanamycin-resistance 0.2% (1/576) and ofloxacin-resistance 0.2% (1/576). The MDR rate was 1.1% (6/576) and no extensively drug resistant tuberculosis (XDR) was detected.

**Conclusions:**

The data show that reorganization of the NTCP resulted in a strong decrease in streptomycin-resistance and suggest that it prevented the emergence of XDR in the Central Region of Cameroon.

## Introduction

Tuberculosis, the second leading cause of death among infectious disease worldwide, is responsible for an estimated 1.4 million deaths each year [1]. Sub-Saharan Africa is the most severely affected area due to the simultaneous occurrence of the tuberculosis and the HIV/AIDS pandemics. According to the 2012 WHO TB report, 80% of the global 0.4 million deaths from HIV/TB coinfection were found in Africa [1]. WHOs recommended approach to tuberculosis-care is the STOP TB Strategy, which is based on Directly Observed Treatment Short-course (DOTS). This approach has been shown to be able to control the spread of drug resistant-tuberculosis in the Americas [2], [3] and Asia [4], [5].

Effective control of tuberculosis is specifically threatened by the emergence of multidrug-resistant (MDR) tuberculosis and extensively drug-resistant (XDR) tuberculosis [6] highly prevalent in Southern Africa [1] [7]. This raises the question of the presence of XDR-tuberculosis in other areas of sub-Saharan Africa, where the number of tuberculosis cases reported might be limited due to infrastructural difficulties.

Cameroon has been classified as a moderate tuberculosis burden country according to the WHO [1]. Two studies conducted at the National Tuberculosis Centre of Jamot Hospital, from 1994 to 1996 assessed the level of resistance in newly diagnosed tuberculosis patients and drug resistance in previously treated tuberculosis patients and found overall resistance rates of 31.8% and 58.2% respectively [8] [9]. Based on these findings, the National Control Tuberculosis Program (NTCP) was reorganized in 1995. The reorganization was introduced in the Central Region in 1998 along with the implementation of the DOTS policy. This reform resulted in a significant increase of cure rates from 45% in 1995 to 70% in 1998 and an impressive drop in the loss-to-follow-up rate from 35% in 1995 to 13% in 2000 [10]. Additionally, since 2004 antituberculosis drugs are delivered free of charge thanks to the support from global health initiatives. As a result of this, detection rate has reached 68% and cure rate has increased up to 67% [10]. Over the past decade, the effectiveness of TB control activities has been evaluated in terms of detection and cure rates reached. A recent WHO report indicated that surveillance of resistance to antituberculosis drugs is an additional means by which to assess the performance of TB control efforts [15]. Thus, the objective of the current study was to assess the level of drug resistance in the Central Region 12 years after the implementation of the NTCP along with the DOTS policy in the same setting.

## Materials and Methods

### Ethics Statement

Ethical clearance and administrative authorization were respectively obtained from the Cameroon National Ethic Committee and the Cameroonian Ministry of Public Health. Signed informed written consent was obtained from each enrolled patient. Patients under 18 years were able to be included into the study provided that an informed consent was signed by their guardians.

### Study Setting and Design

This was a cross-sectional study involving all pulmonary smear positive patients, age ≥15 years attending Jamot hospital from April 2010 to March 2011. All patients underwent physical examination and their histories were recorded. Jamot Hospital, the largest urban reference centre for tuberculosis in the Central Region of Cameroon, is situated in Yaoundé, the capital of Cameroon. This study site is among the 223 diagnostic and treatment centres for tuberculosis patients coordinated by the Cameroon National Tuberculosis Control Program. All the sputa collected from the enrolled patients were evaluated for acid fast bacilli (AFB). Sample processing, confirmatory microscopy, serology, culture, drug susceptibility testing and quality control (internal and external) were performed at the Mycobacteriology laboratory of the Centre Pasteur of Cameroon in Yaoundé (CPC).

A study questionnaire was designed for patient data collection at the study site including gender, age, marital status, educational level, place of residence (urban vs. rural), previous incarceration and clinical data (HIV status, previous TB treatment).

### Sample Processing

Samples were collected from each participant with productive cough on two consecutive days following standard procedures. Ziehl-Neelsen and/or auramine smear examinations [11], [12] were performed at the recruitment site. Only samples with the highest smear grade were transported in a cold box within 8 hours to the CPC for confirmatory microscopy, culture and drug susceptibility testing (DST). Each specimen was submitted to a decontamination step using lauryl sulfate sodium.

HIV testing was performed using a microparticle enzyme immunoassay (AxSYM HIV Ag/Ab Combo) (Abbot Laboratories) for all consenting participants. Each participant received appropriate counselling prior to blood collection.

### Sputum Culture and Identification

After centrifugation, the sediment of the decontaminated product was used to inoculate three Löwenstein Jensen (LJ) media slants, of which one was supplemented with a 0.4% pyruvate. Cultures were incubated at 37°C, read weekly for growth and considered negative when no colony was obtained after 8 weeks of incubation. Final species identification of *M. tuberculosis* was based on growth rate and colony morphology observation. The para-nitrobenzoïc acid (PNB) test was used to distinguish atypical mycobacteria from *M. tuberculosis* complex strains.

### Drug Resistance Assays and Definitions

For all positive cultures, drug susceptibility testing was performed using the indirect proportion method on LJ medium at the following drug concentrations: isoniazid (INH) (0.2 µg/ml and 1 µg/ml), rifampicin (RIF) (40 µg/ml), ethambutol (EMB) (2 µg/ml) and streptomycin (SM) (4 µg/ml), ofloxacin (OFX) (2 µg/ml) and kanamycin (KAN) (20 µg/ml).

Drug resistance was defined as growth on a drug containing medium greater than or equal to 1% for INH and RIF, and 10% for SM and EMB [13]. Resistance to a particular drug without resistance to other anti-TB drugs was defined as “mono-resistance”. Patients were categorized as having, mono-resistance, poly-resistance, multidrug-resistance or extensively drug-resistance according to standard definitions [14], [15]. Resistance to INH and RIF with or without resistance to other anti-TB drugs was defined as “multidrug-resistant tuberculosis” (MDR-TB) (excluding resistance to any second line drug), while “extensively drug-resistant tuberculosis” (XDR-TB) refers to MDR-TB strains additionally resistant to a fluoroquinolone and an injectable agent such as kanamycin, amikacin, viomycin, or capreomycin. “Poly-resistance” was defined as resistance to more than one drug with exception of the combination of INH and RIF.

According to WHO/IUATLD [15], resistance among new cases is defined as the presence of resistant isolates of *M. tuberculosis* in patients who, in response to direct questioning, deny having had any prior anti-TB treatment (for at least 1 month), whereas resistance among previously treated cases is defined as the presence of resistant isolates of *M. tuberculosis* in patients who, in response to direct questioning, admit of having been treated for tuberculosis for 1 month or more.

### Data Analysis

All data were encoded and entered into Epi-info software with restricted access (Centers for Disease Control, Atlanta, USA). Continuous variable (age) was summarized by mean ± SD. Proportions were compared using the chi-square test or the Fischer’s exact test when necessary. Univariate analysis using logistic regression was performed to evaluate the association between drug resistance and socio-demographic and clinical characteristics. p values less than 0.05 were considered to be statistically significant.

## Results

### Characteristics of the Study Population

**Figure 1:**
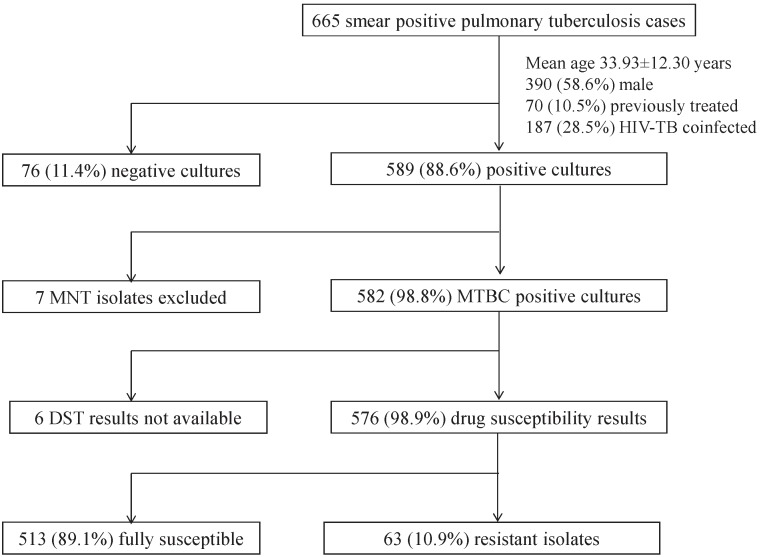
Study population in Jamot hospital, CANTAM survey, Cameroun, 2010–2011.

From April 2010 to March 2011, 665 consecutive smear positive pulmonary tuberculosis cases were enrolled at Jamot Hospital. They were 390 (58.5%) males and 275 (41.5%) females. Patient’s age ranged from 15 to 85 years with a mean of 33.9±12.3 years. Of the 665 cases, 656 (98.7%) consented for HIV testing out of which 187 were HIV infected giving a rate of HIV-TB coinfection of 28.5% (95%CI [25.2–32.1]). A total of 595 (89.5%) cases had no history of TB treatment, whereas 70 (10.5%) had previously undergone anti-tuberculosis treatment. Positive cultures were obtained for 589 (88.6%) sputum specimens. Of these, 7 (1.1%) *Mycobacterium* other than tuberculosis (MOTT) were excluded after identification. The patient data collected and the proportion of positive cultures are shown in Figure 1.

### Overall Resistance in the Study Population

Valid DST results were available for 576 (98.9%) of the 582 strains of *M. tuberculosis* complex (Table 1). Sixty three (10.9%) of the 576 isolates were resistant to at least one drug. Single drug-resistance (mono-resistance) was in found 8.5% (49/576). The most frequent type of single drug-resistance was to INH 4.7% (27/576) followed by SM-resistance 3.3% (19/576) and RIF-resistance 0.2% (1/576). Of the 27 isolates with INH-mono-resistance, 16 displayed low level resistance (INH 0.2 µg/ml) while the remaining 11 showed high level resistance (INH 1 µg/ml). Mono-resistance to KAN and OFX were encountered in 1 case each, which corresponds to a percentage of 0.2% for each drug (Table 1).

**Table 1:** Pattern of resistance among new cases and previously treated cases to anti-tuberculosis drugs among isolates from 576 patients with pulmonary tuberculosis at Jamot hospital, Cameroun, 2010–2011.

Resistance to:	Overall resistance(All cases = 576)n (%)	Resistance among new cases(New cases = 517)n (%)	Resistance among previously treated cases(Previously treated cases = 59)n (%)	p-value
**Mono-resistance**				
INH	27 (4.7)	25 (4.8)	2 (3.4)	
SM	19 (3.3)	16 (3.1)	3 (5.1)	
RIF	1 (0.2)	1 (0.2)	0 (0.0)	
EMB	0 (0.0)	0 (0.0)	0 (0.0)	
KAN	1 (0.2)	1 (0.2)	0 (0.0)	
OFX	1 (0.2)	1 (0.2)	0 (0.0)	
Subtotal	49 (8.5)	44 (8.5)	5 (8.5)	0.99
**Poly-resistance**				
INH-SM	4 (0.7)	2 (0.4)	2 (3.4)	
RIF-SM	1 (0.2)	1 (0.2)	0 (0.0)	
RIF-KAN	1 (0.2)	1 (0.2)	0 (0.0)	
INH-SM-EMB	1 (0.2)	1 (0.2)	0 (0.0)	
INH-OFX	1 (0.2)	1 (0.2)	0 (0.0)	
Subtotal	8 (1.4)	6 (1.2)	2 (3.4)	0.19
**Multidrug-resistance (MDR)**				
INH-RIF	5 (0.9)	3 (0.6)	2 (3.4)	
INH-RIF-SM	1 (0.2)	1 (0.2)	0 (0.0)	
Subtotal	6 (1.1)	4 (0.8)	2 (3.4)	0.11
XDR	0 (0.0)	0 (0.0)	0 (0.0)	
Total	63 (10.9)	54 (10.4)	9 (15.3)	0.26

The overall rate of MDR-tuberculosis cases was 1.1% (6/576). The overall poly-resistance rate found in our study was 1.4% (8/576). No XDR was observed in the entire study population.

### Resistance among New Cases

Among the 576 patients with available DST results, 517 (89.8%) were new cases. A total of 54 (10.4%); (95%CI [8.1%–13.3%]) were resistant to at least one drug. Mono-resistance was encountered in 44 (8.5%) isolates. INH-resistance and SM-resistance were observed in 4.8% (25/576) and 3.1% (16/576) respectively. Of the 25 INH-resistant tuberculosis patients, 10 exhibited high level resistance whereas 15 had low level resistance. Single resistance to EMB was not observed. MDR was detected in 0.8% (4/576) of cases and poly-resistance in 1.2% (6/576).

### Resistance among Previously Treated Cases

Of the 576 patients with valid DST, 59 (10.2%) had previously undergone anti-tuberculosis treatment. Drug resistance was observed in 15.3% (9/59) among which 8.5% (5/59) showed single drug-resistance and 3.4% (2/59) showed MDR and 3.4% (2/59) showed poly-resistance. (Table 1). Single drug SM-resistance was 5.1% (3/59) and INH-resistance was found in 3.4% (2 of 59).

### Risk Factors Associated with Drug Resistance

The overall rate of drug resistance among TB-HIV coinfected patients was 10.5% (17/162) and 11.2% (46/412) among HIV negative patients. Isolates from previously treated patients were more likely to be mono-resistant against SM, however this was not statistical significant. The MDR rate among previously treated cases was higher than among new cases, but this difference was also not statistically significant. Drug resistance tended to be more frequent in individuals older than 25 years of age compared to individuals between 15–24 years of age but this was not statistically significant. No statistical association was noted with regard to education, gender, previous incarceration, place of residence, HIV serostatus and marital status (Table 2).

**Table 2:** Socio-demographic characteristics associated with drug-resistance in smear positive pulmonary tuberculosis patients at Jamot hospital, Cameroun, 2010–2011- Univariate analysis.

Characteristics	N = 576	Resistant isolates		OR [CI95%]	p-value*
		n = 63	%		
**Gender**					
Male	339	38	11.2	1.07 [0.62–1.82]	0.81
Female	237	25	10.5	Ref	
**Marital status**					
Single	394	49	12.4	1.33 [0.81–2.17]	0.25
Married/Ever married	182	14	7.7	Ref	
**Age (years)**					
≥25	433	53	12.2	1.85 [0.91–3.73]	0.08
15–24	143	10	7.0	Ref	
**Previous incarceration**					
Yes	38	6	15.8	0.64 [0.26–1.6]	0.33
No	535	57	10.7	Ref	
**HIV serology** #					
Negative/Non determined	412	46	11.2	1.07 [0.6–1.93]	0.81
Positive	162	17	10.5	Ref	
**Previous TB treatment**					
Yes	59	9	15.3	1.52 [0.71–3.27]	0.28
No/Unknown	517	54	10.4	Ref	
**Education level**					
Illiterate/Primary	197	21	10.7	0.95 [0.54–1.65]	0.85
University/Secondary	379	42	11.1	Ref	
**Place of residence**					
Urban	484	51	10.5	0.78 [0.4–1.54]	0.48
Rural	91	12	13.2	Ref	

# : Only 574 patients consented for HIV testing.

*The p-values assesses statistical significance of difference in resistance among groups defined by gender, marital status, age, previous incarceration, HIV serology, previous TB treatment, education level and place of residence.

## Discussion

Monitoring the magnitude and trends of anti-tuberculosis drug resistance provides an indicator of the quality and performance of the national tuberculosis control program [16]. The purpose of this study was to assess the level of drug resistance in the Central Region of Cameroon 12 years after the reorganization of the NTCP. A previous study has indicated that the prevalence of drug resistance in the Central region of Cameroon might be decreasing [17]. However, this investigation was conducted in relative small numbers of individuals. The present study aims to evaluate the performance of the NTCP by comparing drug resistance levels at the same hospital before and 12 years after reorganization of the NTCP. Prior to the establishment of the NTCP, TB care and management was relatively centralized with referral of TB suspects to specialized centers such as Jamot hospital were diagnosis and care management were performed [10]. The reorganization of the NTCP in the Central Region in 1998 resulted in the creation of 53 TB care management centres named Diagnostic and Treatment Centres (DTC) across the Central Region. In these DTCs trained medical and paramedical staff diagnose TB and administrate standardized treatment according to the WHO endorsed DOTS protocol. Since 2004, thanks to the global health support, antituberculosis drugs are available and delivered free of charge to TB patients in Cameroon [10].

In 1994/95 an investigation of 516 consecutive pulmonary tuberculosis patients at Jamot hospital reported an overall drug resistance rate in new cases of 31.8% [8]. In 1996/97, another study on drug resistance rate among 111 patients with a previous history of tuberculosis therapy revealed a prevalence of 58.2%. In the current investigation the rates for resistance among new cases and previously treated cases were 10.4% and 15.3% respectively. In this context it is important to note that the DST for both investigations were performed at the CPC. The CPC is a member of the international network of Pasteur institutes and as such is subject to regular external quality control.

The decrease in drug resistance among new cases appears to be primarily a consequence of the reduction in SM-resistance from 15.1% in 1994/5 to 3.1% in 2011 and a reduction of poly-resistance from 6% to 1.2%. Prior to 1996, SM was widely used for the treatment of tuberculosis in Cameroon [18]. However, its use as a first line drug was stopped in 1996. The decrease in SM-resistance is therefore likely because the drug is no longer used as a first line drug. To the best of our knowledge this is the first study showing that the removal of SM as a first line drug results in decreased resistance in the *Mycobacterium tuberculosis* complex strains. Recently SM was reintroduced as part of the nationwide retreatment plan during the first two months of retreatment (2 months of SM in addition to RIF, INH, ETH and PZA) [19]. Future SM-sensitivity monitoring will determine if this will have an effect on the SM-resistance rate. The rate for INH-mono-resistance in this investigation was 4.8% which appears to be largely unchanged from the 6% INH-resistance reported in 1994/5. Equally the rate for RIF-mono-resistance of 0.2% was identical between 1994/5 and 2010/11. This suggests that the reorganization of the NTCP was able to prevent an increase of these types of mono-resistance.

Fluoroquinolones (FQ) have been recommended for the treatment of drug sensitive and drug resistant tuberculosis [20]. However, the widespread use of these compounds in the treatment of upper respiratory tract infections raised the concern that this may contribute to the development of FQ-resistance in *M. tuberculosis* [21]. In the current investigation only a single isolate exhibited OFX-mono-resistance, suggesting that OFX-mono-resistance is currently not widespread among *M. tuberculosis* isolates from the Central Region of Cameroon. Among the 8 MDR strains identified in this investigation, none were OFX-resistant. Given the overall low number of MDR strains it is difficult to extrapolate this finding but it suggests that OFX can be used as second line drug in the Central Region of Cameroon.

The prevalence of MDR-tuberculosis in the current study was 0.8% among the new cases. This is essentially unchanged compared to the 0.6% of MDR-tuberculosis observed among new cases in 1994/5. This indicates that even with the implementation of a NCTP along with a DOTS policy it is difficult to decrease the prevalence of initial MDR in a population highlighting the need for second line drug treatment programs.

We also noted a decrease in MDR tuberculosis rate among previously treated cases from 31.8% in 1997/98 to 3.4% in the current study. The relatively low numbers of previously treated patients included in the study resulted in larger 95% confidence interval. However, it is worth pointing out that since 2010 all retreatment sputum samples from the Central, South and East regions of Cameroon are sent to the CPC for culture and DST. If MDR is detected the patient is referred to specialized centers such as Jamot hospital. Therefore it appears unlikely that the decreased rate of MDR among previously treated cases is due to decreased referral of previously treated cases to Jamot hospital. In addition the centralized nature of TB management in 1995 might have resulted in increased loss to follow-up rates [10] possibly contributing to a higher rate of MDR tuberculosis in 1997/98.

No XDR was detected within the entire study population. Together with the absence of MDR strains resistant against all baseline tuberculosis drugs these findings indicate that the reorganization of the NTCP prevented the occurrence of this type of resistant tuberculosis in the Central Region of Cameroon.

In this context it is important to note that a recent retrospective study of retreatment pulmonary tuberculosis in the Littoral Region revealed an overall prevalence of acquired MDR-tuberculosis of 12% [22]. To the best of our knowledge there are no data from the pre-DOTS era available from the Littoral Region but it appears necessary to conduct additional studies in the 10 regions of Cameroon to delineate the national trend with respect to drug-resistant tuberculosis. This also serves as a reminder that point of care methods to detect drug resistant tuberculosis, such as the WHO recommended GeneXpert assay are indicated in areas with a high prevalence of drug resistance in order to enable MDR-TB case detection to initiate second line drug therapy.

In this study, HIV-TB coinfection was observed in 28.4% cases compared to 16.6% reported for the Central Region in 1997 [23]. Although the rate of TB-HIV coinfection was high in our setting, drug resistance was not correlated with HIV infection. This observation is similar to that reported in numerous studies in Sub-Sahara Africa [23], [24]. We recently reported on a small investigation employing spoligotyping method on a subset of the isolates of the current study that showed a predominance of the LAM10_CAM family and a complete absence of the Beijing strains family [25]. Given that the population structure of *M. tuberculosis* strains in high HIV and MDR burden is often dominated by strains of the Beijing family [26], the complete absence of the Beijing family together with the absence of XDR shows that there is currently no evidence for a clonal expansion of the MDR and XDR strains from Southern Africa into Central Africa areas. Future studies using high resolution genotyping techniques such as MIRU-VNTR are necessary to determine the population structure of drug sensitive and drug resistant *M. tuberculosis* strains on isolates from the 10 regions of Cameroon.

The high rate of drug resistance in previously treated patients (15.3%), compared to new cases (10.4%) that was observed in this study is consistent with others studies conducted in sub-Saharan Africa and elsewhere. Previous treatment for tuberculosis has been identified in many settings as an important risk factor for drug resistant tuberculosis acquisition [27]. However, in this study no statistical association was found between previous TB treatment and development of drug resistance. This may be due to the low number of previously treated cases enrolled in this study.

The present study has demonstrated a significant drop in overall SM-resistance and an absence of XDR in the Central Region of Cameroon 12 years after reorganization of the NTCP. These results clearly indicate that efficient control of drug resistant tuberculosis is possible in sub-Saharan Africa despite the dual burden of HIV and tuberculosis.

CANTAM is a network sponsored by the EDCTP with the objective is to conduct baseline investigations on TB, HIV and Malaria in Central Africa in preparation for clinical trials in its members states Gabon, Cameroon and the Republic of Congo [28]. CANTAM is currently conducting a similar investigation in Congo to assess if the trends observed in this study are representative for Central Africa as a whole.
